# Temozolomide plus whole brain radiotherapy for the treatment of non-small-cell lung cancer patients with brain metastases: A protocol of an updated systematic review and meta-analysis

**DOI:** 10.1097/MD.0000000000018455

**Published:** 2020-01-31

**Authors:** Hua Duan, Shu-Yue Zheng, Tian Zhou, Hui-Juan Cui, Kai-Wen Hu

**Affiliations:** a Beijing University of Chinese Medicine; b Oncology Department, Dongfang Hospital, Beijing University of Chinese Medicine; c Department of Integrative Oncology, China-Japan Friendship Hospital, Beijing, China.

**Keywords:** brain metastases, meta-analysis, non-small cell lung cancer, protocol, temozolomide, whole brain radiotherapy

## Abstract

**Introduction:**

Whole brain radiotherapy (WBRT) has been the mainstay treatment of brain metastases (BM) in non-small cell lung cancer (NSCLC) patients for years. Temozolomide (TMZ) could penetrate the blood–brain barrier and some studies showed that TMZ plus MBRT may improve clinical effectiveness. This meta-analysis is aim to evaluate the clinical effectiveness and safety of TMZ plus MBRT in the NSCLC patients with BM.

**Methods and analysis:**

We systematically searched databases including PubMed, EMBASE, Cochrane Central Register of Controlled Trials, and four Chinese databases (Chinese Biomedical Database, China National Knowledge Infrastructure, Wanfang Database and Chinese Scientific Journal Database) without language restrictions from inception until July 26, 2019. Randomized controlled trials (RCTs) which compared TMZ plus WBRT with single WBRT in the advanced NSCLC patients with BM were included. The outcomes analysis reported objective response rate (ORR), disease control rate (DCR), overall survival (OS), progression-free survival (PFS), quality of life (QOL), and adverse effects. Two reviewers will independently extract data from the selected studies and assess the quality of studies. Statistical analyses will be performed using Review manager 5.3 software. Random-effects or fixed models were used to estimate pooled hazard ratio and relative risk.

**Results:**

This systemic review and meta-analysis will evaluate the effects of TMZ plus MBRT in the NSCLC patients with BM in RCTs.

**Conclusion:**

Our study will provide evidence to judge if TMZ plus MBRT are effective treatment for NSCLC patients with BM.

## 1 Introduction

About 20% to 65% of lung cancer patients will occur brain metastases (BM), which is the most common type.^[1–3]^ The total incidence of BM in patients with non-small cell lung cancer (NSCLC) was 15% to 30%.^[4]^ The median survival time was only 1 month without treatment and would extend to 2 months with glucocorticoids^[5]^ which increased an inconceivable burden on society. According to the National Comprehensive Cancer Network (NCCN) guideline, palliative external-beam RT was recommended to treat diffuse BM. Whole brain radiotherapy (WBRT) has been the mainstay treatment of multiple symptomatic BM for years.^[6]^ which could improve headache and motor function by 82% and 61% to 74%, respectively.^[7]^ Although the intracranial response rate of WBRT was 35%,^[7]^ there were still about 1/3 of patients with brain metastasis failed to control intracranial lesions after WBRT, and 50% of patients died from intracranial lesion.^[8]^ Therefore, WBRT combination with other treatments is extremely important.

Temozolomide (TMZ) is a second-generation alkylating agent which could be converted into active alkylating agent precursor in human body and penetrate the blood–brain barrier.^[9]^ A retrospective control study^[10]^ showed that TMZ combined with WBRT could not only improve the intracranial objective response rate (ORR) (34.9% vs 20.2%, *P* = .01), disease control rate (DCR) (98.4% vs 92.7%, *P* = .03) and median progression-free survival (PFS) (5.9 months vs 4.9 months, *P* = .002), but also prevent neurocognitive function (NCF) and quality of life (QOL) from worsening compared with WBRT alone in the treatment of 238 NSCLC patients with BM. Another randomized controlled study^[11]^ recruited 82 NSCLC patient with BM between December 2011 and August 2013, and found that the PFS at 3 and 6 months were 38.46% and 23.77% for WBRT alone vs. 90.70% and 76.43% for TMZ plus WBRT (*P* = .00001).

Up to now, there were two systematic reviews^[12,13]^ focus on the point. One review^[13]^ only included 4 RCTs (published in 2003–2011). Another review^[12]^ included 12 RCTs (published in 2007–2017) which contained some retrospective studies wrongly. Moreover, neither study was registered in PROSPERO or COCHRANE. Owing to these shortcomings, a comprehensive, updated and PRISMA-compliant systematic review^[14]^ of RCTs is necessary to evaluate the effectiveness and safety of TMZ plus WBRT for the NSCLC patients with BM.

## 2 Methods

### 2.1 Registration

This protocol has been registered with the PROSPERO international prospective register of systematic reviews (ID = CRD42019145604). The systematic review and meta-analysis protocol was drafted according to the Preferred Reporting Items for Systematic Reviews and Meta-Analysis Protocols (PRISMA-P) statement.^[15]^

### 2.2 Eligibility criteria

#### 2.2.1 Type of study

The randomized controlled trials (RCTs) compared TMZ plus MBRT and MBRT alone with more than 10 patients enrolled in each group will be included.

#### 2.2.2 Type of participant

Patients who were diagnosed as NSCLC with BM will be included. The age must be 18 years and older and there is no restriction on gender or gene status.

#### 2.2.3 Type of intervention

We will include studies in which intervention group using TMZ plus MBRT as the main intervention, and the control group using MBRT alone.

#### 2.2.4 Types of outcome measurements

##### 2.2.4.1 Primary outcomes

1. ORR (measured by World Health Organization or Response Evaluation Criteria in Solid Tumors criteria)
2. health-related QOL (measured by a validated tool such as the Performance Status, Karnofsky Performance Score, Eastern Cooperative Oncology Group and Quality of Life Questionnaire-Core and so on)

##### 2.2.4.2 Secondary outcomes

1. DCR (measured by World Health Organization or Response Evaluation Criteria in Solid Tumors criteria)
2. overall survival (OS)
3. PFS
4. adverse events (measured by the National Cancer Institute Common Terminology Criteria or World Health Organization criteria.)

#### 2.2.5 Exclusion criteria

Exclusion criteria were the following:

1. case reports, case series, commentaries, reviews, retrospective study, non-control studies, cohort studies, quasi-RCTs and summary,
2. studies with missing or wrong data,
3. studies with anti-tumor treatment besides TMZ and MBRT.

### 2.3 Search methods for primary studies

#### 2.3.1 Electronic searches

The following databases will be searched from their inception to July 26, 2019: PubMed, EMBASE, Cochrane Central Register of Controlled Trials, Chinese Biomedical Database (CBM), China National Knowledge Infrastructure (CNKI), Wanfang Database and Chinese Scientific Journal Database (VIP). No language restriction will be applied.

#### 2.3.2 Search strategy

Following the principle of combining subject words with text words, search strategies in English electronic databases were listed in Table 1, and we will adapt for other resources with appropriate terms.

**Table 1:**
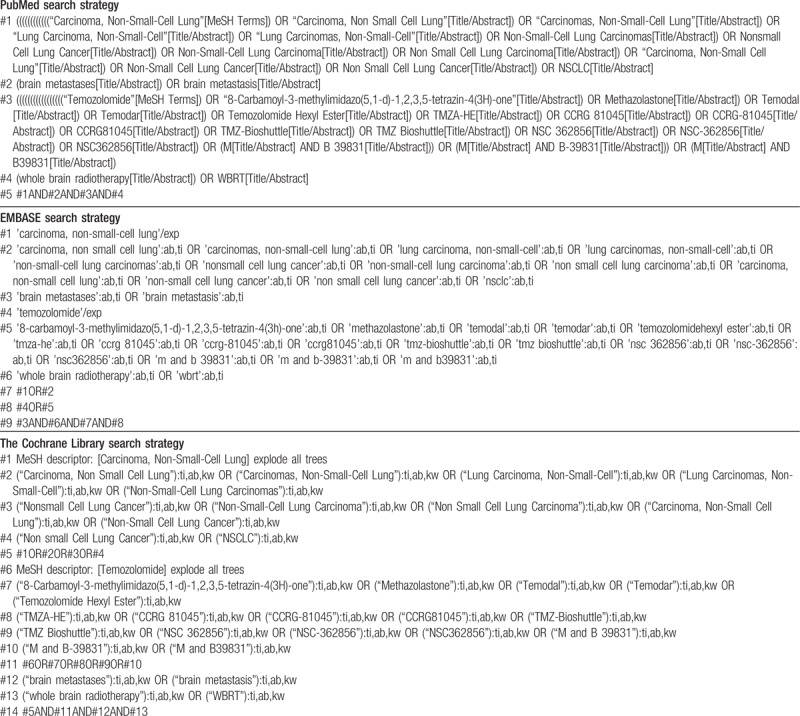
Search strategies for English electronic databases.

### 2.4 Data collection

#### 2.4.1 Selection of studies

**Figure 1:**
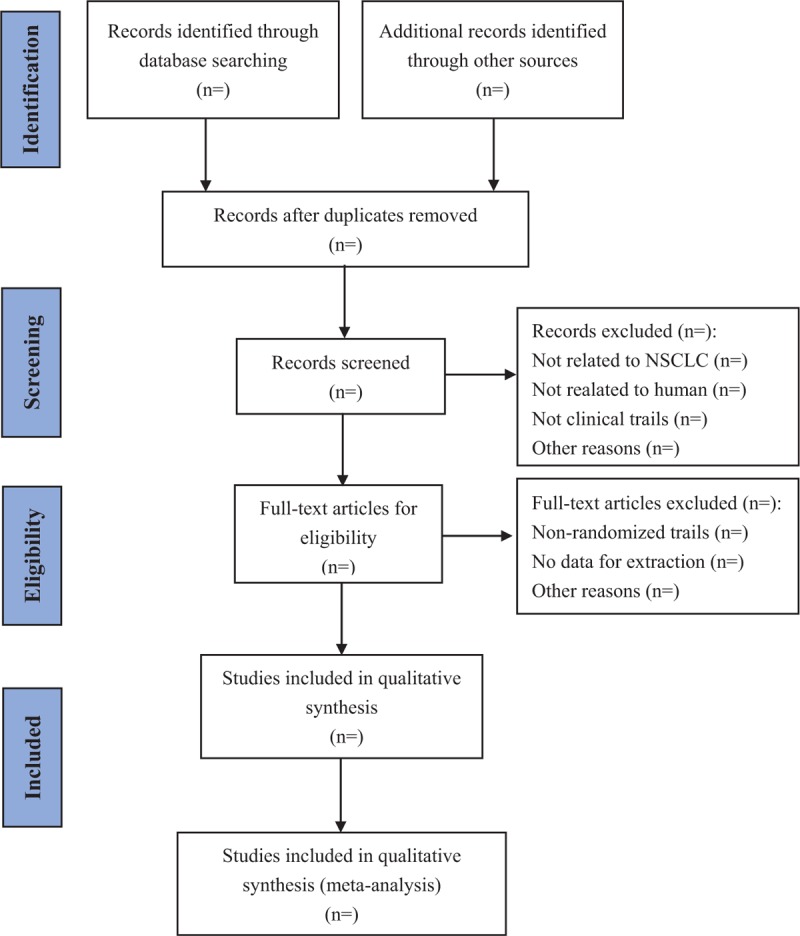
The preferred reporting items for systematic reviews and meta-analyses protocols flow diagram of the study selection process. NSCLC = non-small cell lung cancer.

The retrieved researches will be imported in Endnote software 9.1 to remove duplicates. Two reviewers (HD, SYZ) will independently screen the titles and abstracts of these studies. Potentially eligible studies will be confirmed by evaluating the full text. Any uncertainty or disagreements will be resolved by a third investigator (TZ). The detailed screening process will be shown in the following PRISMA-P flow diagram (Fig. 1).

#### 2.4.2 Data extraction and management

Two reviewers (HD, SYZ) will independently extract data from the selected studies with a predesigned data form. Disagreements will be resolved by discussion or consulting a third reviewer (TZ). Extracted information included years of publication, the first author, sample size, age, gender, TMZ, MBRT, outcome indicators, and so on.

#### 2.4.3 Assessment of risk of bias in included studies

Two reviewers (HD, SYZ) will independently evaluate the risk of bias for each included study using the Cochrane Collaboration's risk of bias tool, consisting of the following items: random sequence generation, allocation concealment, blinding of participants and personnel, blinding of outcome assessment, incomplete outcome data, selective reporting and other bias. We will judge each item as low, high, or unclear risk of bias. Any disagreements will be arbitrated by a third reviewer (TZ).

### 2.5 Data synthesis

Statistical analyses will be performed using Review manager 5.3 software.

#### 2.5.1 Measures of treatment effect

Dichotomous data were expressed as the relative risk (RR). Continuous data were expressed as the weighted mean difference. Survival data were assessed with the hazard ratios (HRs) and 95% confidential intervals (CIs). If we cannot directly extract from the texts, we use Engauge Digitizer (version 4.1) to extract HRs and 95% CIs by computing the Kaplan–Meier graph.^[16]^ A 95% CI was used for all data analysis.

#### 2.5.2 Management of missing data

We will seek missing or insufficient data by contacting the original authors whenever possible. If we failed to obtain the missing data, those dates will be excluded from the analysis.

#### 2.5.3 Assessment of heterogeneity

A Cochran's *Q* test with *P* > .10 and an *I*^2^ of no more than 50% indicated that statistical homogeneity was acceptable. We used a fixed-effect model. Otherwise, we used a random-effect model. On the other hand, if meta-analysis was not feasible because of significant statistical heterogeneity, we will conduct a descriptive analysis.

#### 2.5.4 Subgroup analysis

If the data are available and sufficient, we will conduct subgroup analysis to determine the potential cause of heterogeneity. The different dosage and comparative interventions and so on will be divided into subgroups for analysis according to the actual conditions.

#### 2.5.5 Sensitivity analysis

If there is significant statistical heterogeneity, we will use sensitivity analysis to find the reason by eliminating each study one by one.

#### 2.5.6 Publication bias

Publication biases will be checked by funnel plots generated from data of more than 10 studies.

## 3 Ethics and dissemination

Ethics approval is not required, as this study will not involve patients. The results of this study will be submitted to a peer reviewed journal for publication, to inform both clinical practice and further research.

## 4 Discussion

NSCLC is the most common type of lung cancer which counts about 86%^[17]^ and the incidence of adenocarcinoma, squamous cell carcinoma and large cell carcinoma were 11%, 6%, and 12%, respectively according to Surveillance, Epidemiology, and End Results,^[18]^ which create a serious challenge. NCCN guideline recommend palliative external-beam RT as the standard treatment of BM. WBRT was the most commonly used therapy with increased survival by 2 to 5 months.^[5]^ However, WBRT can only delay the emergence of new intracranial lesions by 0.5 to 1 year, and some patients even develop new intracranial metastases in the process of WBRT.^[8]^ Systemic therapy after WBRT is an important prerequisite for long-term survival.^[19]^

TMZ is a second-generation alkylating agent which can cross the blood–brain barrier to enhance anti-tumor effect in the brain tumor.^[9,20]^ Studies have found that when BM occur, the blood-brain barrier has been partially destroyed, and WBRT can also open the blood-brain barrier.^[21]^

A meta-analysis published in 2018^[12]^ indicated that the combination therapy could prolong the median survival compared to WBRT alone without HR, but some retrospective and non-randomized studies were included wrongly. So the results were unprecise and the effectiveness of TMZ plus WBRT is still unclear. We will conduct this systematic review to evaluate the effectiveness and safeties of TMZ plus WBRT in the treatment of NSCLC patients with BM following the Cochrane Handbook for Systematic Reviews of Interventions to provide different perspectives and guidance for doctors.

## Acknowledgments

None.

## Author contributions

**Conceptualization:** Hua Duan, Shu-Yue Zheng, Kai-Wen Hu, Hui-Juan Cui.

**Data curation:** Hua Duan, Shu-Yue Zheng.

**Formal analysis:** Hua Duan, Shu-Yue Zheng.

**Investigation:** Hua Duan, Shu-Yue Zheng, Tian Zhou.

**Methodology:** Hua Duan, Shu-Yue Zheng.

**Project administration:** Kai-Wen Hu, Hui-Juan Cui.

**Resources:** Hua Duan, Shu-Yue Zheng.

**Software:** Hua Duan, Shu-Yue Zheng.

**Supervision:** Kai-Wen Hu, Hui-Juan Cui.

**Validation:** Hua Duan, Shu-Yue Zheng, Tian Zhou

**Writing – original draft:** Hua Duan, Shu-Yue Zheng

**Writing – review & editing:** Hua Duan, Shu-Yue Zheng, Tian Zhou
